# Popular Contraceptive Methods in Women Aged 35 Years and Older Attending Health Centers of 4 Cities in Khuzestan Province, Iran

**DOI:** 10.5812/ircmj.4414

**Published:** 2013-10-05

**Authors:** Sedighe Nouhjah, Elham Amiri, Azim Khodai, Azar Yazdanpanah, Maryam Nadi Baghu

**Affiliations:** 1 Health Research Institute, Diabetes Research Center, Ahvaz Jundishapur University of Medical Sciences, Ahvaz, IR Iran

**Keywords:** Contraception, Community Health Center, Pregnancy Unplanned

## Abstract

**Background:**

The prevalence of unintended pregnancy and associated risks are higher in late reproductive years. Limited studies have focused on contraceptive choices in these women. The aim of the study was to identify contraceptive choices and their related factors in women 35 years or older attending health centers of Khuzestan province.

**Objectives:**

Additionally, several line of evidence indicated relationship between increasing maternal age and poor pregnancy outcomes (1, 2). Pregnancies above the age of 35 are accompanied with more risks for complication related to pregnancy as compared to younger women (3-5). Risk of spontaneous abortion is 74.4% in mothers aged 45 years or more.

**Patients and Methods:**

In a cross-sectional study 1584 women aged 35 years and older attending public health centers of four cities of Khuzestan were studied. We used an interviewer-administered questionnaire for data collection. Women investigators were recruited for interview and filling the questionnaire. Participants were assured of the confidentiality of their responses.

**Results:**

The mean age of women was 39.8 ± 4.2 years. The most popular contraceptive methods used in this age group were oral contraceptive pills (31.4%), condom (28.1%), and tubal ligation (14.8%). Less effective contraceptive methods were used in 41.5% of women. Significant associations were found between the use of effective methods and literacy of husband (OR = 0.80, 95% CI: 0.75, 0.91), city of residence (OR = 0, 92, 95%CI: 0.87-0.97), women age (OR = 0.97, 95% CI; 0.94-0.99), and women education (OR = 0.87, 95%CI: 0.76-0.99) (P < 0.01).

**Conclusions:**

In spite of risk of pregnancy and unintended pregnancy in this age group, about a half of them used less effective contraceptive methods, hence family planning education, and counseling to older women should be a priority in health centers.

## 1. Background

Unintended pregnancy is a widespread global health problem. Although fecundity is reduced in late years of reproduction, rate of unintended pregnancy among women aged 35 and older still remained high (3). More than thirty percent of pregnancies in women older or equal to 35 years are unintended (6). About 51% of all pregnancies in women aged 40 and older are unintended, and in this age group more than other age groups, about 65% of unintended pregnancy end to abortion (7). One of the highest ratio of induced abortion is reported in women aged 40 years and older (3, 8, 9). Unintended pregnancy in older women is associated with higher risks. In women aged 35-39 years pregnancy-related mortality ratio is twice in comparison to women aged 25-29 years, and women aged 40 and older have a five times greater ratio (6). Maternal mortality is reported 4 times more in mothers aged 35 years and older. Spontaneous abortion rate increases to 26% in mothers aged 40-49 years old (10).

## 2. Objectives

Additionally, several line of evidence indicated an association between increasing maternal age and poor pregnancy outcomes (1, 2). Pregnancy above the age of 35 is accompanied with more risks for complications related to pregnancy as compared to younger women (3-5). Risk of spontaneous abortion is 74.4% in mothers aged 45 years and more (11). Older age of mother in pregnancy is associated with an increased risk of prenatal mortality, risk of chromosomal abnormalities, and still birth (3, 10). Use of Contraceptive method decreases the proportion of unintended pregnancy and associated risks. Women older than 35 years are more likely to forget contraception use than women aged 20-24. Although sterilization is still the most popular chosen contraception for women older 35, in recent years using hormonal methods or withdrawal and rhythm methods is increasing in this age group. No contraception use is reported by about 20% of women aged 40-44 and 15% of women aged 35-39 (3, 6). Contraceptive choices among younger women have investigated in many previous studies, but studies on the pattern of contraception in late years of reproduction are limited.

Therefore using an effective contraceptive method is very important in this group. The overall aim of this study was to assess contraceptive choices and related factors in women aged 35 and older attending health centers of south western of Iran.

## 3. Patients and Methods

In a descriptive- analytic study 1584 married women aged 35-50 years attending public health centers of four cities of Khuzestan province (south western of Iran) were studied.

Four large cities of Khuzestan, Ahvaz, Dezful, Khoramshahr, and Izeh were selected due to having a wider representation of the province. The populations of these cities are about one million, 400000, 150000 and 170000 respectively. The study was performed between October 2009 and December 2010. Every woman aged equal or greater than 35 years seeking family health services (routine care for their children, contraception) during the study period was asked to participate in the study. The sample size was calculated based on a pilot study performed in Ahvaz city (11).

We used an interviewer-administered questionnaire for data collection. Women investigators were recruited for interviewing and filling the questionnaire. Bachelors of public health were recruited as research assistants. Participants were assured of the confidentiality of their responses. We entered and analyzed data through SPSS (version 11.5) software. Chi square and logistic regression tests were performed. The dependent variables were dichotomized (use or nonuse of effective methods) for logistic regression analyses. The level of significance was set at 0.05.

## 4. Results

384 women from Ahvaz city, 700 from Dezful, 200 from Khoramshahr, and 300 from Izeh city completed the study. The mean age of women was 39.8 ± 4.2 years. 26.3% had experienced 1-3 pregnancies and 24.1% of them experienced 5 or more pregnancies in their reproductive ages. Experience of at least one abortion was reported in 19.7% of women. Three most popular methods used in this age group were oral contraceptive pills (31.4%), condom (28.1%), and tubal ligation (14.8%). Less effective contraceptive methods (natural, rhythm and condom) were used in 41.5% of women. Vasectomy in 1.6% of participants was chosen as the contraceptive method. Highly educated women were less likely to use effective methods (37.9% vs. 77.5% in illiterate women) (P < 0.001). Higher use of more effective methods (hormonal, IUD, and surgical) was reported in women with marriage younger than 18 years (69.7%), and parity equal or more than 6 (79.5%) (Table 1). 

**Table 1.** Reproductive Characteristic of Participants

Variable	Number	Percent
**Gravida**		
1	108	6.9
2	401	25.3
3	416	26.3
4	278	17.6
5	174	11.0
≥ 6	207	13.1
Total	1584	100
**Parity**		
1	126	8.0
2	470	29.7
3	422	26.7
4	266	16.8
5	147	9.3
≥ 6	151	9.5
Total	1582	100
**History of Abortion**		
Yes	312	19.7
No	1272	80.3
Total	1584	100

Binary logistic regression showed significant associations between using effective contraceptive methods and educational level of women and their husbands, city of residence, and women age (Table 2, Table 3, Table 4). 

**Table 2.** Contraceptive Methods in Women Aged 35 and Older Attending Health Centers of Four Cities in Khuzestan Province

Contraceptive Method	Number	Percent
**Natural**	208	13.2
**Condom**	445	28.2
**LD**	444	28.1
**Mini Pill**	18	1.1
**Tri Phasic**	37	2.3
**IUD**	130	8.2
**Injection**	36	2.3
**TL**	235	14.9
**Vasectomy**	25	1.6
**Rhythm**	3	0.2
**Total**	1581	100

**Table 3.** Use of Effective Contraceptive Methods (Hormonal, IUD, Surgical) and Less Effective Methods (Natural, Rhythm and Condom) Based on Women Characteristics

Variable	Effective Contraceptive Methods No., (%)	Less Effective Contraceptive Methods No., (%)	Significance Level
**Age**			< 0.001
35 - 39	503 (55.8)	398 (44.2)	
40 – 57	414 (62.2)	252 (37.8)	
**Marriage age**			< 0.001
≤ 18	315 (69.7)	137 (30.3)	
18 – 24	440 (57.4)	327 (42.6)	
25 – 29	129 (46.7)	147 (53.3)	
≥ 30	39 (47.6)	43 (52.4)	
**Educational Level of Women**			< 0.001
Illiterate	141 (77.5)	41 (22.5)	
Primary	215 (70.0)	92 (30.0)	
Intermediate	229 (64.7)	125 (35.3)	
High School	232 (51.0)	223 (49.0)	
Collage	106 (37.9)	174 (62.1)	
**Literacy of Husband**			< 0.001
Illiterate	95 (77.2)	28 (22.8)	
Primary	174 (69.0)	78 (31.0)	
Intermediate	237 (67.5)	114 (32.5)	
High School	289 (57.3)	215 (42.7)	
Collage	130 (37.1)	220 (62.9)	
**City of Residence**			< 0.001
Dezful	382 (54.8)	315 (45.2)	
Khoramshahr	129 (64.5)	71 (35.2)	
Izeh	208 (69.3)	92 (30.7)	
Ahwaz	206 (53.6)	178 (46.4)	
**Gravidity**			< 0.001
1	35 (32.7)	72 (67.3)	
2	184 (45.9)	217 (54.1)	
3	229 (54.6)	188 (54.4)	
4	186 (66.9)	92 (33.1)	
5	131 (75.3)	43 (24.7)	
≥ 6	163 (78.7)	44 (21.3)	
**Parity**			< 0.001
1	47 (37.6)	78 (62.4)	
2	211 (450)	258 (55.0)	
3	239 (56.8)	182 (43.2)	
4	191 (71.8)	75 (28.2)	
5	117 (79.6)	30 (20.4)	
≥ 6	120 (79.5)	31 (20.5)	
**History of Abortion**			NS
Yes	188 (60.5)	123 (32.5)	
No	737 (58.0)	533 (42.0)	

**Table 4.** Results of Regression Logistic 95% Confidence Interval

Variable	Odds Ratio	CI	Signification Level
**Women Education**	0.87	0.76-0.99	0.04
**Husband’s Literacy**	0.82	0.72-0.94	0.004
**City of Residence**	0.92	0.87-0.97	0.007
**Gravida**	1.27	0.92-1.7	NS
**Parity**	1.17	0.85-1.6	NS
**Women’s Age**	0.97	0.94-0.99	0.03

## 5. Discussion

In spite of a decline in fecundity in late years of reproduction, the prevalence of unintended pregnancy and related complication in pregnancy in this age group is high. In the present study 3 most popular contraceptive methods used by women aged 35 years and older were OCP (31.5%), condom (28.1%), and tubal ligation (14.9%). Similar to our results Musharrafi and colleagues (2003) reported OCP as the most popular method in this age group (30%) (12). In many prior studies surgical methods were reported as the most popular contraceptive methods in this age group of women in developed countries (13). Because many clients did not return to health centers after referring for surgical methods, our estimation about these methods may be underestimated. In contrast to our finding, Abma et al. reported that only 11% of women aged 35-39 years and 6% of women aged 40-44 years used old combination of OCP. They reported that concerns about safety of OCP in these age groups lead to low use of this method (14). Less effective methods (natural, rhythm and condom) were used in 41.5% of participants. Frost and Darroch reported that women aged 35-44 years were more likely to use natural, condom, and other barrier methods (15). Studies indicated that in spite of choosing sterilization as a common contraceptive method, hormonal methods or withdrawal and rhythm were chosen by women aged 35 years and older in recent years (16, 17). Our study had some limitations. Populations of the study were women attending health centers and may be not an actual representation of women in this age group. We estimated most popular methods used by women in this study, but we missed women who did not use any methods in this age group. Recent studies reported higher rate of contraceptive nonuse in this age group (3, 17, 18). A relative big sample size, and focus on this specific age group were the strengths of this study.

In spite of risk of pregnancy and unintended pregnancy in this age group, about a half of them used less effective contraceptive methods. Health centers should take extra care to counsel women of this age group. Counseling about hazards of pregnancy in these years and also non contraceptive benefits of hormonal methods should be a priority in health centers.
